# Eustachian Tube Function Assessment after Radiofrequency Turbinate Reduction in Atopic and Non-Atopic Patients

**DOI:** 10.3390/ijerph18030881

**Published:** 2021-01-20

**Authors:** Francesco Martines, Francesco Dispenza, Federico Sireci, Salvatore Gallina, Pietro Salvago

**Affiliations:** 1 Bi.N.D. Department, University of Palermo, Via del Vespro, 129, 90127 Palermo, Italy; pietrosalvago@libero.it; 2 Istituto Euromediterraneo di Scienza e Tecnologia—IEMEST, Via M. Miraglia 20, 90139 Palermo, Italy; francesco.dispenza@gmail.com; 3 ENT Department, A.O.U.P. Paolo Giaccone, Via del Vespro, 129, 90127 Palermo, Italy; federicosireci@hotmail.it (F.S.); salvatore.gallina@unipa.it (S.G.)

**Keywords:** eustachian tube dysfunction (ETD), chronic nasal obstruction, turbinate hypertrophy, ETDQ-7

## Abstract

(1) Background: Inferior turbinates’ hypertrophy is often associated with Eustachian tube dysfunction (ETD); radiofrequency turbinate reduction (RTR) may provide a long-term improvement of nasal obstruction and ETD-related symptoms. (2) Aim: The study aimed to compare ETD in atopic and non-atopic patients before and after RTR and to investigate the correlation between tympanometry and Eustachian Tube Dysfunction Questionnaire-7 (ETDQ-7). (3) Methods: Ninety-seven patients, ranging from 33 to 68 years old, were screened by skin tests and divided into atopic (G1) and non-atopic (G2). Eustachian tube function (ETF) was evaluated through tympanometry, William’s test and ETDQ-7. (4) Results: A moderate to severe subjective ETDQ-7 was found in the 35.42% of G1 and in the 22.45% of G2 patients before RTR. William’s test resulted normal in 141 ears (72.68%), partially impaired in 15 (7.73%), and grossly impaired in 38 (19.59%) before surgery. A grossly ETD was evidenced in the 19.59% of cases before surgery and decreased to 6.18% after surgery with a significant difference among atopic patients (*p* < 0.001). (5) Conclusion: RTR may be considered a treatment option in patients suffering from ETD and inferior turbinates’ hypertrophy; RTR reduced the percentage of grossly impaired ET function (*p* < 0.001). ETDQ-7 and William’s test may represent valuable tools to assess ET function before and after surgery.

## 1. Introduction

Chronic nasal obstruction due to inferior turbinate hypertrophy is one of the most frequent ENT complaints in patients suffering from allergic and non-allergic rhinitis [1,2,3,4]; it is often associated with fullness of one or both ears, earache, tinnitus and hearing impairment. All these symptoms may be correlated to Eustachian tube dysfunction (ETD); in fact, the ET connects the middle ear with the nasopharynx, equalizing pressure between the tympanic cavity and atmospheric pressure and draining secretions from the middle ear to the nasopharynx [5,6,7,8]. ETD has an estimated prevalence ranging from 0.9% to 4% in the adult population and has been suggested as a causal factor in different ear pathologies [9,10,11].

Intranasal steroids, antihistamines, chromones and sympathomimetics are usually prescribed to relieve symptoms related to enlargement of the inferior turbinate, but in non-responsive patients, turbinate surgery (radiofrequency ablation, diode laser and microdebrider-assisted inferior turbinoplasty) should be suggested [12,13,14,15,16,17].

It is widely demonstrated that inferior turbinate surgery maximizes volumetric reduction of the turbinate and may provide a long-term improvement of nasal obstruction; however, data about the effect of turbinate surgery on ETD and hearing related symptoms are still scarce [18,19,20,21].

Manometric tests study ventilatory and pressure equalization abilities of the ET. One of the simplest manometric tests is tympanometry, which is the basic test in case of suspected ETD; it provides an indirect measure of ET function (ETF) by measuring middle ear pressure.

If the ET is functioning normally, middle ear pressure should be equal to atmospheric pressure, with a −20 to +20 decapascal (daPa) range in the 95% of healthy subjects [22]. However, many studies have found middle ear pressure to be slightly negative even in healthy ears and pressures from −50 to +50 daPa can be considered normal in adults [8,23,24]. This test remains particularly effective in detecting middle ear effusions [2,4,6], with a reported sensitivity and specificity of 94% and 95%, respectively [25].

Different methods were described to assess ET function using manometric tests. The easiest uses basic tympanometry equipment to look at patient-induced pressure changes in the middle ear while the patient performs a forcible ‘sniff’, a Valsalva or a Toynbee maneuver; however, Doyle et al. reported low percentage values of sensitivity and specificity [26].

Other common manometric tests are the Toynbee’s test and automated ETF–William’s test used in both perforated and intact tympanic membrane and had a reported accuracy of 81% [27]. All the other methods proposed to assess ET function (e.g., sonotubometry, tubomanometry, nasopharyngeal maneuvers, video-endoscopy, Electromyography) still have low percentage values of sensitivity (ranging from 74.2% to 87%) and specificity (ranging from 65.6% to 67%) but are also limited by the cost of equipment and the evidence that aural complaints are not always correlated to tympanometry [10,11,12,27,28,29].

For this reason, in 2012 McCoul et al. introduced the Eustachian Tube Dysfunction Questionnaire-7 (ETDQ-7) for quantitative assessment of ETD-related symptoms with an ‘Ideal’ of sensitivity and specificity of 100%; this validated organ-specific tool consists of seven questions with a seven-item Likert scale, with a response of 1 indicating no problem and 7 indicating a severe problem and a final score ranging from 7 to 49 points [13,14,27,30,31].

The aim of this study was to compare ETD in atopic and non-atopic patients before and after inferior turbinate surgery and to investigate the correlation between tympanometry measurements and ETDQ-7 scores.

## 2. Materials and Methods

This study was an observational cohort study involving 113 adult patients recruited from September 2017 to September 2019. All subjects included complained chronic nasal obstruction and at least one aural symptom (fullness or clogging of the ears, earache, tinnitus and inability to rapidly compensate middle ear pressure) which did not respond to a 3-month trial of appropriate treatment (topical corticosteroids, antihistamines, and/or sympathomimetics). Through examination by anterior rhinoscopy and nasal endoscopy, an experienced otolaryngologist confirmed the presence of bilateral inferior turbinate hypertrophy and referred the patient for radiofrequency turbinate reduction (RTR). Patients with significant nasal septum deviation, internal/external valve collapse/stenosis, chronic rhinosinusitis with or without polyposis, sinonasal and nasopharyngeal tumors, previous nasal and/or ear surgery, severe systemic disorder and severe obesity were excluded.

The study protocol was fully explained and written informed consent was obtained from each patient. Approval for this study had been obtained from the local ethical committee (Approval No. 28/06).

Skin tests were performed using 12 common perennial and seasonal allergens: *Alternaria, Aspergillus, Cladosporium, Penicillium*, ragweed, grass mix, trees mix, cockroach, dust mites, *Dermatophagoides farinae* and *Dermatophagoides pteronyssinus*, and cat and dog epithelium. Solutions of histamine and saline were used as positive and negative controls, respectively. The results were evaluated after 10 min. Wheals ≥3 mm in diameter than wheals at the site of the negative control were considered positive. The subjects with at least 1 positive skin prick test to any antigen were classified as atopic [17] and included in the group 1 (G1) while those with negative skin prick test (non-atopic) were included in the group 2 (G2).

All surgical procedures were performed by the same surgeon using a digital 130 Watt mono/bipolar unit with an operating frequency of 4 MHz and a modulation frequency of 33 kHz.

The procedures were carried out under local anesthesia with patients’ eyes covered. First, the inferior turbinates were topically anesthetized using cotton strips with a mixture of lidocaine 20 mg/mL and 2–3 drops of epinephrine 0.1% in 5 to 10 mL of lidocaine. The local anesthetic (10 mg/mL lidocaine hydrochloride with 2 to 3 mL epinephrine—Lidocain) was then applied through a 24-gauge needle into the anterior and medial parts of the inferior turbinate.

The Binner bipolar needle electrode was inserted submucosally in the inferior half of the turbinate longitudinally; the first entry was in the mid anterior part of the inferior turbinate, and the additional two entries were performed medially introducing the needle as far as the posterior end of the turbinate. The parts were treated for 10 s at 35-watt (W) output power in all three areas. Because submucosally (intraturbinally) delivered radiofrequency energy in the inferior turbinate creates a focal lesion with no damage to adjacent structures (e.g., turbinate bone or mucosal surface), the patients were allowed to leave the office without medication after two hours of the completion of the treatment; they were also advised to use ibuprofen or ketoprofen for local pain or, to contact the center if needed, and do not use oral or topical steroids, antihistamines, or decongestants during the follow-up period.

All patients were evaluated two weeks before the surgery and after three months by the surgery; during the visits, the subjects filled the ETDQ-7 for quantitative assessment of ETD-related symptoms before meeting the examiner.

The ETDQ-7, a validated organ-specific tool, consists of seven questions with a seven-item Likert scale, with a response of 1 indicating no problem and 7 indicating a severe problem and a final score range from 7 to 49 points (total score <14.5 is considered normal) [30,31]. We decided to divide the total score by 7 to give an overall score ranging from 1.0 to 7.0. This equated to an ETDQ-7 mean item score of ≥2.1 to indicate the presence of ETD [18].

Pure tone audiometry (PTA) was performed before surgery by a trained audiologist with an Amplaid 309 audiometer in a soundproof audiometric room. Air conduction was measured on-ear TDH-49 headphones set for 250–8000 Hz; bone conduction was measured using a calibrated bone transducer for 250–4000 Hz. Mean PTA resulted as 24.15 dB HL. No cases of conductive and mixed hearing loss were detected.

The Amplaid 766tympanometer, with a probe frequency of 220 Hz and an air pressure range of −400–100 daPa with automatic recording, was used for tympanometry and to study ET function. Tympanograms were divided into the following types: type A (+99 to −99 daPa), type C (>−100 daPa) and type B (flat curve without peak identification).The tympanometry measurements considered were: ear canal volume (ECV), that is an estimation of the volume of air medial to the probe, which includes the volume between the probe tip and the tympanic membrane; the tympanometry peak pressure (TTP), corresponding to ear canal pressure at which the peak of the C tympanograms occurs; the static compliance (SC), that describes the greatest amount of acoustic energy absorbed by the middle ear system.

ET function was tested through William’s test in which the impedance audiometer is programmed to measure the middle ear pressure in 3 consecutive conditions: at the start of the test (resting pressure—Peak 1), after the patient swallows (with the nose and mouth closed—Peak 2), and finally after performing Valsalva (Peak 3). Normally, the ambient (i.e., resting) middle ear pressure should be at or near the atmospheric air pressure (i.e., approx. 0 mm of water pressure); the tympanic cavity pressure should become negative on swallowing and positive on performing Valsalva. The findings of the ETF-William’s test for the different groups of patients were classified into 3 categories: (1) Perfectly normal function, (2) Partially impaired function and (3) Grossly impaired function. To evaluate the functionality of the Eustachian tube, we used the Peak 1-Peak 2 > 20 daPa (after swallow) and Peak 3-Peak 1 > 20 daPa (after Valsalva) criteria. If the ambient middle ear pressure becomes negative on swallowing but does not become positive on Valsalva or vice versa the function is considered to be partially impaired. If the middle ear pressure does not change either after swallowing and after Valsalva maneuver, the ET function is considered grossly impaired.

Statistical analysis was conducted performing χ^2^ test, odds ratio (OR), correlation analysis (r) and *t* test, following usual conditions of application. Significance was set at 0.05.

## 3. Results

Out of 113 patients, 5 subjects were excluded because of severe systemic disorders diagnosed during the admission analysis; a total of 108 patients underwent surgery but finally 97 (mean age = 48.2 ± 9.1) were included in the study because 11 patients were treated with antihistamines associated to selective leukotriene receptor antagonists during follow-up period. The age range was 33–68 years with a mean age of 48.22 ± 9.04.

Based on skin tests, they were classified in 2 groups: G1 (atopic) and G2 (non-atopic), consisting of 48 and 49 subjects, respectively.

Among G1 group, 22 patients (45.83%) had positive skin tests for both inhalant and food allergens; 9 patients (18.75%) had a positive test only for food allergy; 17 (35.42%) patients had an allergy only against inhalant allergens.

**Figure 1:**
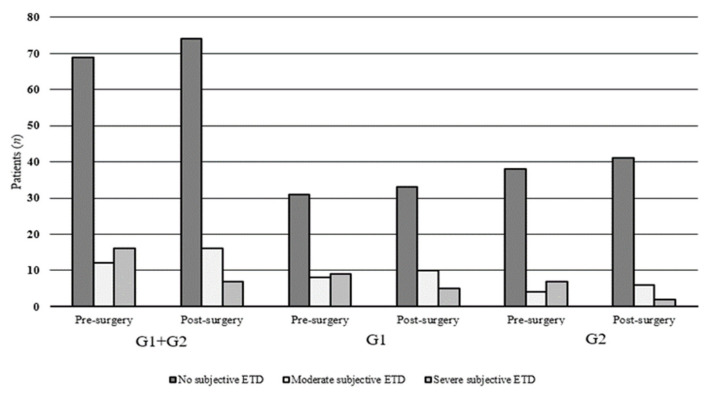
Eustachian Tube Dysfunction Questionnaire-7 (ETDQ-7) among atopic and non-atopic patients.

Sixty-nine patients had a preoperative ETDQ-7 total score of 1.0 to 1.9 (without subjective dysfunction), 12 presented a 2.0 to 2.9 score (moderate ETD) and 16 had a score >3.0 (severe ETD) with a range of 3.0–3.7 (Figure 1). The distribution of ETDQ-7 total score among the groups evidenced a ETD in 35.42% of atopic (8 subjects with a score of 2.0 to 2.9 while 9 with a score >3.0) and in the 22.45% of non-atopic patients (4 subjects with a score of 2.0 to 2.9 and 7 with a score >3.0) preoperatively. The percentage of patients with a normal preoperative ETDQ-7 resulted respectively 64.58% and 77.55% for G1 and G2 groups without a statistically significant difference (*p* = 0.319) (Figure 1). 

The study of the mean values of single ETDQ-7 items showed higher mean scores in atopic respect to non-atopic patients; a significant difference was found among atopic subjects between pre and post-surgery only in case of the following question: ‘A feeling that your ears are clogged?’ (*p* = 0.049); no difference was found regarding the other items (*p* > 0.05) (Table 1).

Among tympanometry measurements TTP ranges from −62 to +39 daPa and from −60 to +41 daPa respectively for G1 and G2 evidencing a type “A” tympanogram for all patients (*p* > 0.05).

The ECV and the SC mean values for the total ears resulted in being 1.73 ± 0.69 cc and 1.18 ± 0.37 cc with a significant difference between the groups (G1: ECVmean value = 1.85 ± 0.62 cc; SC mean value 1.30 ± 0.28 cc; G2: ECV mean value = 1.60 ± 0.72 cc; SC mean value: 1.06 ± 0.41 cc; *p* < 0.01) (Table 2). In the 42.78% of cases for ECV and in the 6.63% of patients for SC the results were out of normal range with a higher percentage inside the atopic group (*p* < 0.01).

Out of 194 ears examined, the William’s test resulted normal in 141 cases (72.68%), partially impaired in 15 (7.73%) and grossly impaired in 38 (19.59%). The distribution among the groups evidenced a significant difference with an ET normal function in the 79.59% of G2 group with respect to the 65.62% of G1 group (*p* = 0.003) (Table 3).

In the 81.25% of patients with a severe ETDQ-7 score a grossly impaired ET function was evidenced while 94.96% of subjects with a normal ETDQ-7 presented a normal ET function (*p* < 0.001) with a correlation index r = 0.99 (Table 3).

The ETDQ-7 filled by patients three months after surgery showed an improvement with a 76.29% of normal ET function (74 cases, 33 G1 and 41 G2), a 16.49% with a moderate ETD (16 cases, 10 G1 and 6 G2) and a 7.22% (5 G1 subjects and 2 G2) reporting severe ETD (*p* = 0.014). Also, the distribution of the mean values of single ETDQ-7 items showed an improvement between pre- and post-surgery related to G1, G2 and total cohort but without any significant difference (*p* = 0.1) (Table 1).

At follow up examination we observed an improvement of ECV (1.50 ± 0.46 cc) and SC (1.17 ± 0.28 cc) in most of cases; in fact, the percentage values out of normal range decreased to 26.80% and to 4.12% for ECV and SC, respectively. A significant difference was evidenced for ECV (*p* < 0.0001).The differences in tympanometry measurement between G1 and G2 groups were also confirmed three months after surgery (G1: ECV mean value =1.60 ± 0.49 cc; SC mean value 1.23 ± 0.29 cc; G2: ECV mean value = 1.39 ± 0.38 cc; SC mean value: 1.11 ± 0.25 cc; *p* = 0.002) (Table 2).

William’s test results changed significantly during follow up examination; we noted a reduction of ‘grossly ET impaired function’ that decreased from 19.59% to 6.18% respect to a higher percentage of ‘partial impaired function’ that increased from 7.73% to 21.65% (*p* < 0.001). This statistical difference was more evident inside G1 population where ‘grossly ETD’ decreased from 29.17% to 8.33% and ‘partial impaired function’ percentage changed from 5.21% to 26.04% (*p* < 0.001).

As seen before the surgery, also three months after surgery the distribution of William’s test and ETDQ-7 scores in the cohort resulted as being statistically significant (*p* < 0.001); in fact, the 84.45% of subjects with a normal ETF presented a normal ETDQ-7 with a correlation index r = 0.87.

## 4. Discussion

The relationship between the middle ear ventilation, ET, and nasal cavities has been the subject of numerous studies [2,3,5,9,11,15,25,32,33,34,35]; Low and Willatt in 1993 evaluating the relationship between middle ear pressure and a deviated nasal septum observed an improvement of TTP in both ears after septal surgery [32]; the authors postulated that postnasal airflow turbulence associated with a deviated nasal septum could lead to ETD. It could be explained because several patients, either atopic or non-atopic, usually complain to suffer from middle ear ventilation problems concomitant with nasal obstructive pathologies [1,2,6,14,28,32,33,34]. Certainly, all authors agreed that ETD is thought to be an important cause of middle ear disturbances [1,2,3,4,5,6,7,8,25,32,35]. Despite an initial pharmacological treatment, patients with inferior turbinates’ hypertrophy and nasal obstruction often undergo turbinate radiofrequency ablation [12,13,14,15,16,19]. This surgery usually provides good long-term results but the changes on ET function and improvement of the ear related symptoms are usually not investigated or partially evaluated using only tympanometry [18,19,20,21,32,33].

In 2012 McCoul et al. introduced the Eustachian Tube Dysfunction Questionnaire-7 (ETDQ-7), now universally recognized as a good tool to identify people with subjective and objective ETD [29,30].

The percentage of abnormal tympanometry measurements and altered William’s test among patients suffering from chronic nasal obstruction varies with a range from 12.5% to 58% [36,37]. In line with Lazo-Saenz et al. (2005), who reported either doubled percentages of ETD and higher ECV and SC mean values in atopic cohort with respect to control group, we observed a grossly ETD in the 29.17% of G1 group with respect to the 10.20% of G2 cohort, and a higher value of ECV and SC out of normal range in G1 respect to G2 (56% vs. 29.59% for ECV and 10.41% vs. 3.06% for SC) [38].

Some authors demonstrated that surgery improved tympanometry and ET function, while others reported a not significant improvement in ET function postoperatively [32,33,34,36,37]. In line with Salvinelli et al. who demonstrated an improved ET function after nasal surgery, we observed a significant reduction of ‘grossly ETD’; in fact, out of 38 ears with grossly ETD (28 ears G1 and 10 ears G2), only the 6.18% (12 ears: 8 belonging to G1 and 4 to G2) did not show any improvement after surgery (*p* < 0.001) [35]. Moreover, among tympanometry measures, there was a significant normalization of ECV average during the follow-up (*p* < 0.001).

In a study conducted by Doğan et al., 80 patients with six different septum types and consequent nasal obstruction underwent tympanometry measurement and Automated ETF (based on William’s test) using Impedance Audiometry AC40 before and 6 months after surgery [39]. The author considered the ‘Peak 1-Peak 2 > 10 daPa or Pmax-Pmin > 15 daPa’ criteria to study ETF; all measurements improved 6 months after surgery and when the obstruction was localized in the inferior meatus and in the floor of nasal cavity the improvement resulted significant. Using the same criteria, we obtained similar results after RTR; comparing pre- and post-surgery Peak 1-Peak 2 and Peak 3-Peak1 mean values, we demonstrated how volumetric reduction of the turbinates improved significantly the William’s test results (*p* < 0.001).

Therefore, RTR could be considered an option for those subjects who complain ETD associated to chronic nasal obstruction due to inferior turbinate hypertrophy with or without atopy.

Even if ETDQ-7 was validated in 2012, only from 2017 it was used by Harju et al. to study the effect of inferior turbinate surgery on ETD-related symptoms [18,30,31]. The authors showed a statistically significant improvement in all subjects, either after real surgeries that were limited to the anterior part (*p* = 0.03 for radiofrequency ablation and *p* = 0.006 for microdebrider-assisted inferior turbinoplasty) and after sham surgery (*p* = 0.04). For this reason, Harju et al. concluded that reduction of the anterior half of the inferior turbinate, as the only procedure to treat the ear symptoms, is useless because the operation does not involve the posterior part of the turbinate, which is close to the ET orifice.

In our cohort, the ETDQ-7 questionnaire filled before surgery resulted reliable; in fact, in the 81.25% of a severe ETDQ-7 score it was evidenced a grossly impaired ET function at William’s test and the 94.96% of subjects with a normal ETDQ-7 had a normal ET function (*p* < 0.001). Compared to Harju et al. who observed before surgery a normal ETDQ-7 in 45% of patients, we had higher normal values (71.13%) especially among non-atopic patients (77.55%) that usually had less hearing-related complains. This is reasonable because for three months before surgery all patients underwent to oral and/or topical nasal therapy that could have improved aural symptoms present ‘ab origine’. The ETDQ-7 reliability was also confirmed after surgery where a normal ETDQ-7 was found in the 84.45% of subjects with a normal ET function (r = 0.87; *p* < 0.001).

The ETDQ-7 mean value decreased from 1.8 before surgery to 1.6 at follow up (*p* = 0.12); it overlaps to the normal ETF percentage at William’s test between pre (72.68%) and post-surgery (72.16%) that unchanged. However, it should not be interpreted as a failure to treat ETD with RTR; in fact, grossly impaired ETF has dropped (*p* = 0.014) and the ETF (Peak 1-Peak 2: r = 0.89; Peak 3-Peak 1) improved significantly both in atopic and non-atopic patients.

This study presents the following limitations: to begin with, all patients were assessed only once at three months after surgery, thus we could not be able to rule out fluctuations of ETF during the 3-month period or in the long-term. Secondly, the study sample is relatively small, and results are difficult to compare because data regarding RTR and ETD are still scarce.

## 5. Conclusions

The present study confirmed the close relationship between aural symptoms and chronic nasal obstruction due to inferior turbinate hypertrophy. In particular, atopic patients presented a higher prevalence of ETD (34.38% vs. 20.40%, *p* = 0.044) and a higher ETDQ-7 (35.42% vs. 22.45%, *p* = 0.15) score respect to non-atopic subjects.

RTR may be considered an option in chronic ETD with inferior turbinates’ hypertrophy which does not respond to medical therapy; in fact, as highlighted by our results, surgical treatment reduced the percentage of grossly impaired ETF (*p* < 0.001), normalized the ECV average (*p* < 0.001) and increased ETF both in atopic and non-atopic patients.

With a correlation index of r = 0.99 before and of 0.87 after surgery (*p* < 0.001), ETDQ-7 and William’s test may represent valuable tools to assess both subjectively and objectively candidates for RTR.

## Figures and Tables

**Table 1:** Eustachian Tube Dysfunction Questionnaire-7 (ETDQ-7) scores before and after surgery.

ETDQ-7	Atopic + Non-Atopic G1 + G2			Atopic G1			Non-Atopic G2		
Items	Pre-Surgery Mean (SD)	Post-Surgery Mean (SD)	T-Test (*p*-Value)	Pre-Surgery Mean (SD)	Post-Surgery Mean (SD)	T-Test (*p*-Value)	Pre-Surgery Mean (SD)	Post-Surgery Mean (SD)	T-Test (*p*-Value)
Pressure in the ears?	1.75 ± 0.81	1.55 ± 0.79	0.09	1.87 ± 0.87	1.72 ± 0.89	0.41	1.63 ± 0.75	1.38 ± 0.63	0.08
Pain in the ears?	1.87 ± 1.37	1.64 ± 1.10	0.21	2.12 ± 1.52	1.89 ± 1.29	0.43	1.63 ± 1.18	1.41 ± 0.83	0.28
A feeling that your ears are clogged or “under water”?	1.94 ± 0.96	1.69 ± 0.89	0.05	2.25 ± 0.88	1.87 ± 0.95	0.04	1.65 ± 0.95	1.51 ± 0.79	0.42
Ear symptoms when you have a cold or sinusitis?	2.21 ± 1.20	1.97 ± 1.01	0.14	2.41 ± 1.25	2.23 ± 1.03	0.42	2.02 ± 1.14	1.73 ± 0.93	0.18
Crackling or popping sounds in the ears?	1.61 ± 0.99	1.47 ± 0.84	0.31	1.73 ± 1.04	1.58 ± 0.91	0.47	1.49 ± 0.93	1.36 ± 0.75	0.48
Ringing in the ears?	1.27 ± 0.55	1.26 ± 0.54	0.89	1.29 ± 0.50	1.27 ± 0.49	0.84	1.26+0.60	1.26 ± 0.60	1
A feeling that your hearing is muffled?	1.78 ± 0.89	1.64 ± 0.75	0.25	1.83 ± 0.86	1.68 ± 0.77	0.38	1.73 ± 0.93	1.61 ± 0.73	0.47
Mean Total Score (Min–Max)	12.46 ± 5.77 (8–26)	11.26 ± 5.04 (7–26)	0.12	13.52 ± 6.11 (8–26)	12.27 ± 5.61 (7–26)	0.29	11.42 ± 5.28 (8–25)	10.28 ± 4.25 (7–21)	0.24
Mean Item Score (Min–Max)	1.8 ± 0.8 (1.1–3.7)	1.6 ± 0.7 (1.0–3.7)	0.12	1.9 ± 0.9 (1.1–3.7)	1.7 ± 0.8 (1.0–3.7)	0.29	1.6 ± 0.7 (1.1–3.5)	1.5 ± 0.6 (1.0–3.0)	0.24

**Table 2:** William’s test and tympanometry measurements before and after surgery.

William’s Test	Atopic + Non-Atopic			Atopic			Non-Atopic		
	G1 + G2			G1			G2		
	Ears (194)			Ears (96)			Ears (98)		
	Pre-surgery	Post-surgery	χ^2^	Pre-surgery	Post-surgery	χ^2^	Pre-surgery	Post-surgery	χ^2^
	***n*** (%)	***n*** (%)	(*p*-value)	***n*** (%)	***n*** (%)	(*p*-value)	***n*** (%)	***n*** (%)	(*p*-value)
Normal	141 (72.68)	140 (72.16)	(0.0001)	63 (65.62)	63 (65.62)	(0.0001)	78 (79.59)	77 (78.57)	
Partially Impaired	15 (7.73)	42 (21.65)		5 (5.21)	25 (26.04)		10 (10.20)	17 (17.35)	
Toymbe	7	15		2	10		5	5	
Valsalva	8	27		3	15		5	12	0.1
Grossly Impaired	38 (19.59)	12 (6.18)		28 (29.17)	8 (8.33)		10 (10.20)	4 (4.08)	
Tympanometry measurements			T-Test			T-Test			T-Test
			(*p*-value)			(*p*-value)			(*p*-value)
ECV (cc)									
Mean ± SD	1.73 ± 0.69	1.50 ± 0.46	(0.0001)	1.85 ± 0.62	1.60 ± 0.49	(0.002)	1.60 ± 0.72	1.39 ± 0.38	(0.01)
SC (cc)									
Mean ± SD	1.18 ± 0.37	1.17 ± 0.28	(0.72)	1.30 ± 0.28	1.23 ± 0.29	(0.10)	1.06 ± 0.41	1.11 ± 0.25	(0.31)

ECV: ear canal volume; SC: static compliance.

**Table 3:** ETDQ-7 score and William’s test assessment before and after surgery.

	William’s Test					
ETDQ-7		Pre-Surgery			Post-Surgery	
	Normally	Partially Impaired	Grossly Impaired	Normally	Partially Impaired	Grossly Impaired
1.0–1.9 No subjective ETD	132	6	0	125	23	0
2.0–2.9 Moderately subjective ETD	7	5	12	13	14	5
>3.0 Severe subjective ETD	2	4	26	2	5	7
TOTAL	141	15	38	140	42	12

Correlation index (r)—pre-surgery: r = 0.99; post-surgery: r = 0.87. ETDQ-7 pre- and post-surgery: *p* = 0.014. William’s Test pre- and post-surgery: *p* < 0.001. ETD: Eustachian tube dysfunction.

## Data Availability

The data are not publicly available due to privacy restrictions.

## References

[1] Pelikan Z. (2006). Chronic otitis media (secretory) and nasal allergy. Scr. Med..

[2] Bentivegna D., Salvago P., Agrifoglio M., Ballacchino A., Ferrara S., Mucia M., Sireci F., Martines F. (2012). The linkage between Upper Respiratory Tract Infections and Otitis Media: Evidence of the ‘United airways concept’. Acta Med. Medit..

[3] Tewfik T.L., Mazer B. (2006). The links between allergy and otitis media with effusion. Curr. Opin. Otolaryngol. Head Neck Surg..

[4] Martines F., Salvago P., Ferrara S., Messina G., Mucia M., Plescia F., Sireci F. (2016). Factors influencing the development of otitis media among Sicilian children affected by upper respiratory tract infections. Braz. J. Otorhinolaryngol..

[5] Casselbrant M.L., Mandel E.M., Rosenfeld R.M., Bluestone C.D. (2000). Epidemiology. Evidence-Based Otitis Media.

[6] Martines F., Bentivegna D. (2011). Audiological investigation of Otitis Media in Children with Atopy. Curr. Allergy Asthma Rep..

[7] Mucia M., Salvago P., Brancato A., Cannizzaro C., Cannizzaro E., Gallina S., Ferrara S., La Mattina E., Mulè A., Plescia F. (2015). Upper respiratory tract infections in children: From case history to management. Acta Med. Medit..

[8] Sade J., Ar A. (1997). Middle ear and auditory tube: Middle ear clearance, gas exchange, and pressure regulation. Otolaryngol. Head Neck Surg..

[9] Browning G.G., Gatehouse S. (1992). The prevalence of middle ear disease in the adult British population. Clin. Otolaryngol. Allied Sci..

[10] Ferrara S., Di Marzo M., Martines F., Ferrara P. (2011). Medical and surgical update on “atelectasic-Adhesive-Tympanosclerotic” otitis media. Otorinolaringologia.

[11] Bluestone C.D. (2005). Eustachian Tube. Structure, Function, Role in Otitis Media.

[12] Hillas J., Booth R.J., Somerfield S., Morton R., Avery J., Wilson J.D. (1980). A comparative trial of intranasal beclomethasone dipionate and sodium chromoglycate in patients with chronic perennial rhinitis. Clin. Allergy.

[13] Kirkegaard J., Mygind N., Molgaard F., Grahne B., Holopainen E., Malmberget H., Brøondbo K., Røjne T. (1987). Ordinary and high-dose ipratropium in perennial nonallergic rhinitis. J. Allergy Clin. Immunol..

[14] Krause H. (1992). Antihistamines and decongestants. Otolaryngol. Head Neck Surg..

[15] Berger G., Gass S., Ophir D. (2006). The histopathology of the hypertrophic inferior turbinate. Arch. Otolaryngol. Head Neck Surg..

[16] Willatt D. (2009). The evidence for reducing inferior turbinates. Rhinology.

[17] Fokkens W.J., Lund V.J., Hopkins C., Hellings P.W., Kern R., Reitsma S., Toppila-Salmi S., Bernal-Sprekelsen M., Mullol J., Alobid I. (2020). European Position Paper on Rhinosinusitis and Nasal Polyps 2020. Rhinology.

[18] Harju T., Kivekäs I., Numminen J., Rautiainen M. (2018). The effect of inferior turbinate surgery on ear symptoms. Laryngoscope.

[19] Li K.K., Powell N.B., Riley R.W., Troell R.J., Guilleminault C. (1998). Radiofrequency volumetric tissue reduction for treatment of turbinate hypertrophy: A pilot study. Otolaryngol. Head Neck Surg..

[20] Elwany S., Gaimaee R., Fattah H.A. (1999). Radiofrequency bipolar submucosal diathermy of the inferior turbinates. Am. J. Rhinol..

[21] Coste A., Yona L., Blumen M., Louis B., Zerah F., Rugina M., Peynègre R., Harf A., Escudier E. (2001). Radiofrequency is a safe and effective treatment of turbinate hypertrophy. Laryngoscope.

[22] British Society of Audiology (2013). Tympanometry: Recommended Procedure.

[23] Seifert M.W., Seidemann M.F., Givens G.D. (1979). An examination of variables involved in tympanometric assessment of Eustachian tube function in adults. J. Speech Hear. Disord..

[24] Shanks J., Shelton C. (1991). Basic principles and clinical applications of tympanometry. Otolaryngol. Clin. N. Am..

[25] Shekelle P., Takata G., Chan L., Mangione-Smith R., Corley P.M., Morphew T., Morton S. (2002). Diagnosis, Natural History, and Late Effects of Otitis Media with Effusion. Evid. Rep./Technol. Assess..

[26] Doyle W.J., Swarts J.D., Banks J., Casselbrant M.L., Mandel E.M., Alper C.M. (2013). Sensitivity andspecificity of eustachian tube function tests in adults. JAMA Otolaryngol. Head Neck Surg..

[27] Smith M.E., Tysome J.R. (2015). Tests of Eustachian tube function: A review. Clin. Otolaryngol..

[28] Liu P., Su K., Zhu B., Wu Y., Shi H., Yin S. (2016). Detection of Eustachian tube openings by tubomanometry in adult otitis media with effusion. Eur. Arch. Otorhinolaryngol..

[29] Van der Avoort S.J., van Heerbeek N., Zielhuis G.A., Cremers C.W. (2005). Sonotubometry: Eustachian tube ventilatory function test: A state-of-the-art review. Otol. Neurotol..

[30] McCoul E.D., Anand V.K., Christos P.J. (2012). Validating the clinical assessment of eustachian tube dysfunction: The Eustachian Tube Dysfunction Questionnaire (ETDQ-7). Laryngoscope.

[31] Teixeira M.S., Swarts J.D., Alper C.M. (2018). Accuracy of the ETDQ-7 Questionnaire for Identifying Persons with Eustachian Tube Dysfunction. Otolaryngol. Head Neck Surg..

[32] Low W.K., Willatt D.J. (1993). The relationship between middle ear pressure and deviated nasal septum. Clin. Otolaryngol. Allied Sci..

[33] Nanda M.S., Kaur M., Bhatia S. (2018). Impact of septoplasty on hearing and middle ear function. Int. J. Res. Med. Sci..

[34] Şereflican M., Yurttaş V., Oral M., Yılmaz B., Dağlı M. (2015). Is middle ear pressure affected by nasal packings after septoplasty?. J. Int. Adv. Otol..

[35] Kaya M., Dağlı E., Kırat S. (2018). Does Nasal Septal Deviation Affect the Eustachian Tube Function and Middle Ear Ventilation?. Turk. Arch. Otorhinolaryngol..

[36] Salvinelli F., Casale M., Greco F., D’Ascanio L., Petitti T., Di Peco V. (2005). Nasal surgery and Eustachian tube function: Effects on middle ear ventilation. Clin. Otolaryngol..

[37] Akyildiz M.Y., Özmen Ö.A., Demir U.L., Kasapoğlu F., Coşkun H.H., Basut O.I., Siğirli D. (2017). Impact of septoplasty on Eustachian tube functions. J. Craniofac. Surg..

[38] Lazo-Sáenz J.G., Galván-Aguilera A.A., Martínez-Ordaz V.A., Velasco-Rodríguez V.M., Nieves-Rentería A., Rincón-Castañeda C. (2005). Eustachian tube dysfunction in allergic rhinitis. Otolaryngol. Head Neck Surg..

[39] Doğan R. (2019). The Effect of Types of Nasal Septum Deviation on the Eustachian Tube Function. Bezmialem Sci..

